# Upregulation of microRNA-96-5p is associated with adolescent idiopathic scoliosis and low bone mass phenotype

**DOI:** 10.1038/s41598-022-12938-3

**Published:** 2022-06-11

**Authors:** Huanxiong Chen, Kenneth Guangpu Yang, Jiajun Zhang, Ka-yee Cheuk, Evguenia Nepotchatykh, Yujia Wang, Alec Lik-hang Hung, Tsz-ping Lam, Alain Moreau, Wayne Yuk-wai Lee

**Affiliations:** 1 Department of Spine Surgery, The First Affiliated Hospital of Hainan Medical University, Haikou, Hainan China; 2 Department of Orthopaedics and Traumatology, SH Ho Scoliosis Research Laboratory, Joint Scoliosis Research Centre of the Chinese University of Hong Kong and Nanjing University, The Chinese University of Hong Kong, Hong Kong SAR, China; 3 Viscogliosi Laboratory in Molecular Genetics of Musculoskeletal Diseases, Sainte-Justine University Hospital Research Center, Montreal, QC Canada; 4 Department of Stomatology, Faculty of Dentistry, Université de Montréal, Montreal, QC Canada; 5 Department of Biochemistry and Molecular Medicine, Faculty of Medicine, Université de Montréal, Montreal, QC Canada; 6 Li Ka Shing Institute of Health Sciences, The Chinese University of Hong Kong, Hong Kong SAR, China

**Keywords:** Biomarkers, Diseases

## Abstract

Bone densitometry revealed low bone mass in patients with adolescent idiopathic scoliosis (AIS) and its prognostic potential to predict curve progression. Recent studies showed differential circulating miRNAs in AIS but their diagnostic potential and links to low bone mass have not been well-documented. The present study aimed to compare miRNA profiles in bone tissues collected from AIS and non-scoliotic subjects, and to explore if the selected miRNA candidates could be useful diagnostic biomarkers for AIS. Microarray analysis identified miR-96-5p being the most upregulated among the candidates. miR-96-5p level was measured in plasma samples from 100 AIS and 52 healthy girls. Our results showed significantly higher plasma levels of miR-96-5p in AIS girls with an area under the curve (AUC) of 0.671 for diagnostic accuracy. A model that was composed of plasma miR-96-5p and patient-specific parameters (age, body weight and years since menarche) gave rise to an improved AUC of 0.752. Ingenuity Pathway Analysis (IPA) indicated functional links between bone metabolic pathways and miR-96-5p. In conclusion, differentially expressed miRNAs in AIS bone and plasma samples represented a new source of disease biomarkers and players in AIS etiopathogenesis, which required further validation study involving AIS patients of both genders with long-term follow-up.

## Introduction

Adolescent Idiopathic Scoliosis (AIS) is the most common form of three-dimensional spinal deformity in paediatric population, which affects adolescent between 10 and 16 years of age with a global prevalence of 2–4%^1^. Due to the unclear etiopathogenesis, the current treatment strategy for AIS is mainly targeting on anatomical abnormality instead of the cause^2^, which often results in suboptimal treatments and psychological sequelae. Therefore, early diagnosis and prediction of curve progression are important research questions to aid patient stratification at earlier stage for more effective and timely treatment.

The observation of discordant curve type and curve severity on monozygotic twins implies the significant role of non-genetic factors in AIS pathogenesis^3^. In the past decade, genome-wide associations studies (GWAS) have reported hundreds of AIS susceptibility loci^4^. Subsequent functional studies revealed role of these genes in etiopathology of AIS, such as ladybird homeobox 1 (*LBX1*) in paraspinal muscle^5–8^. More recent studies from literature suggest that epigenetic mechanisms may contribute to etiopathology of AIS^9^, including DNA methylation, long non-coding RNAs (lncRNA) and microRNAs (miRNAs). DNA methylation changes the activity of genes by adding methyl groups to the DNA molecules with the DNA sequences unchanged^10^. Methylation of Wnt family member 10A (*WNT10A*)^11^, neuropeptide Y (*NPY*)^11^, nestrogen receptor 1 (*ESR1*)^12^, estrogen receptor 2 (*ESR2*)^13^, protocadherin 10 (*PCDH10*)^14^, cartilage oligomeric matrix protein (*COMP*)^15^, hyaluronan synthase 2 (*HAS2*)^16^, pituitary homeobox 1 (*PITX1*)^17^, histone H3 lysine 9 (*H3K9*)^18^, and necdin (*NDN*)^19^ genes were reported to be relevant to AIS. lncRNAs, transcripts longer than 200 nucleotides, do not contain functional open reading frames but regulate gene expression^20^. H19 lncRNA was reported to be differentially expressed in convex and concave side of paravertebral muscle in AIS^21^. Liu et al. have identified 139 lncRNAs that were differentially expressed in the AIS patients when compared with the controls^22^. miRNAs are a class of endogenous short noncoding RNAs (~ 22–26 nucleotides in length) that regulate diverse biological processes^23^ through binding to messenger RNA (mRNA) targets. The miRNAs are known to modulate the sporadic and phenotypical alterations by transducing environmental stimuli into biological signaling^24^. The clinical use of miRNAs is gaining a lot of interests in molecular medicine because they are detectable in many different biological fluids^25^. Emerging evidence suggest the presence of differential miRNA profile in AIS. Abnormal circulating levels of miR-151a-3p^26^, miR-30e^27^, miR-122-5p, miR-27a-5p, miR-223-5p, miR-671-5p and miR1306-3p have been discovered and validated in blood samples from AIS patients in previous case–control studies. Those studies provide strong evidence supporting potential role of epigenetics in AIS^28^.

Osteopenia, defined as a Z-score of femoral neck areal bone mineral density (aBMD) below − 1, was reported in about one third of AIS patients, 80% of which could persist to skeletal maturity^29–31^. Recent studies with high resolution quantitative computed tomography (HR-pQCT) revealed significant alterations of cortical and trabecular bone qualities in AIS with normal BMD by dual energy X-ray absorptiometry (DXA)^32,33^. Cortical volumetric BMD (vBMD) at distal radius was found to enhance prediction of curve progression in AIS^34^. AIS patients had high levels of serum bone turnover markers, and high level of bone turnover in time-lase analysis of local bone formation and resorption was shown to be associated with high risk of curve progression in AIS^35^. Histomorphometry and fluorescent imaging studies suggested larger osteoid area, less osteocyte, aberrant structure and function of osteocyte lacuna-canaliculi network in AIS bone tissues^36,37^, which were partly attributed to differential expression profile of osteogenic genes in AIS osteoblasts^38,39^. The molecular changes underlying these abnormalities could be a source of novel biomarkers.

Our recent study on bone tissues and patient-derived cell culture demonstrated a causative relationship between high miRNA-145-5p expression and abnormal osteocyte function in AIS^41^. In light of the close association between low bone mass and curve progression, we hypothesized that studying the miRNA profile in AIS bone tissues could lead to identification of novel miRNA candidates that are relevant to abnormal bone phenotypes in AIS. This study consisted of three parts: (1) we identified differentially expressed miRNAs by performing miRNA microarray on bone tissues collected from AIS and non-scoliotic subjects; (2) then, a retrospective case–control replication cohort was analysized to determine the plasma levels of the selected miRNA candidates in AIS and healthy subjects; and (3) finally, logistic regression was applied to construct composite diagnostic models for AIS.

## Results

### Clinical and demographic characteristics of participants

Our initial discovery cohort was composed of four severe AIS patients and four age- and ethnically matched non-scoliotic subjects who underwent spinal fusion surgery and orthopaedic bone related reconstructive surgery, respectively. Both groups were recruited for collection of iliac crest bone tissues to identify miRNA candidates by miRNA microarray (Table 1 and Supplementary Table 1). This selection strategy for non-scoliotic subjects with low bone mass being the control group here was pivotal to distinguish miRNAs that were associated with AIS from those linked solely to low BMD. In validation cohort, one hundred AIS girls (with Cobb angles ranging from 15° to 80°) and fifty-two healthy girls were recruited. Anthropometric information, aBMD from DXA and bone quality parameters from HR-pQCT were summarized in Table 2. These two groups were similar to each other in terms of age, body height, arm span, sitting height, Tanner stage of pubic hair and year since menarche. AIS subjects were shown to have significantly lower body weight and slightly earlier Tanner breast stage. DXA results showed significantly lower aBMD at femoral necks in AIS girls than healthy girls. At distal radius, HR-pQCT analysis showed significantly lower cortical area, total vBMD, cortical vBMD, cortical thickness, trabecular vBMD, trabecular BV/TV and trabecular number with higher trabecular separation in AIS. These observations were comparable to previous studies^32^.

**Table 1:** Clinical and demographic characteristics of participants involved in discovery cohort.

	AIS (N = 4, Females)		Non-scoliotic subjects (N = 4, 3 Males and 1 Female)		P value
	Mean	SD	Mean	SD	
Age	15.17	1.60	14.00	3.67	0.580
Standing height (cm)	158.35	5.49	161.63	5.68	0.069
Body weight (kg)	54.23	17.29	53.50	25.30	0.724
Arm span (cm)	158.93	6.38	161.30	6.39	0.275
Cobb angle (°)	48.00	5.89	–	–	–
Tanner stage (pubic hair)	4.00	1.10	3.20	1.64	0.375
Tanner stage (breast)	4.17	0.41	3.80	1.30	0.763

**Table 2:** Clinical and demographic characteristics of participants involved in validation cohort.

	AIS girls (N = 100)	Healthy girls (N = 52)	P value
Age^b^	14.4 (13.6, 15.4)	14.8 (13.5, 15.8)	0.299
Maximum Cobb angle^a^	31.88 ± 13.55		
**Anthropometric measurements**			
Body weight (kg)^a^	43.7 ± 5.9	49.5 ± 10.3	** < 0.001**
Body height (cm)^a^	156.3 ± 5.7	156.2 ± 7	0.915
Arm span (cm)^a^	156.2 ± 6.8	154.1 ± 7.4	0.084
Sitting height (cm)^b^	84 (81.6, 85.5)	83.5 (80.9, 86.5)	0.750
**Maturity assessment**			
Tanner stage (breast)^b^	3 (3, 4)	4 (3, 4)	**0.048**
Tanner stage (pubic hair)^b^	3 (3, 4)	3 (3, 4)	0.552
Year since menarche^a^	2.3 ± 1.1	2.8 ± 1.8	0.083
**DXA parameters**			
Left femoral neck aBMD (g/cm^2^)^a^	0.697 ± 0.067	0.827 ± 0.174	** < 0.001**
Right femoral neck aBMD (g/cm^2^)^a^	0.706 ± 0.079	0.837 ± 0.18	** < 0.001**
**HR-pQCT parameters**			
Total area (mm^2^)^a^	185.1 ± 32.6	180 ± 31.4	0.358
Cortical area (mm^2^)^b^	37.7 (26.2, 46.2)	44.9 (30.2, 55.4)	**0.007**
Trabecular area (mm^2^)^a^	143.6 ± 32.8	132.8 ± 30.9	0.051
Total vBMD (mg/HAmm^3^)^b^	279.9 (238.8, 329.5)	319.7 (253.1, 379.3)	**0.004**
Cortical vBMD (mg/HAmm^3^)^b^	763.3 (703.6, 828.7)	814.1 (741.1, 861.1)	**0.014**
Cortical thickness (mm)^a^	0.64 ± 0.26	0.78 ± 0.30	**0.005**
Cortical perimeter (mm)^a^	55.3 ± 5.0	54.7 ± 4.9	0.447
Trabecular vBMD (mg/HAmm^3^)^a^	136.2 ± 26.1	151.2 ± 35.9	**0.009**
Trabecular BV/TV^a^	0.113 ± 0.022	0.126 ± 0.03	**0.009**
Trabecular number (mm^−1^)^a^	1.575 ± 0.247	1.66 ± 0.281	0.056
Trabecular thickness (mm)^b^	0.072 (0.066, 0.077)	0.075 (0.065, 0.084)	0.148
Trabecular separation (mm)^b^	0.562 (0.508, 0.633)	0.526 (0.466, 0.598)	**0.021**

^a^Normally distributed data were presented as mean ± standard deviation (SD) and independent samples t test was used.

^b^Data that were not normally distributed were presented as median (lower quartile, upper quartile). Mann–Whitney U test was used. Bold text indicated p value < 0.05

### Differential miRNA profile in AIS bone tissues

**Figure 1:**
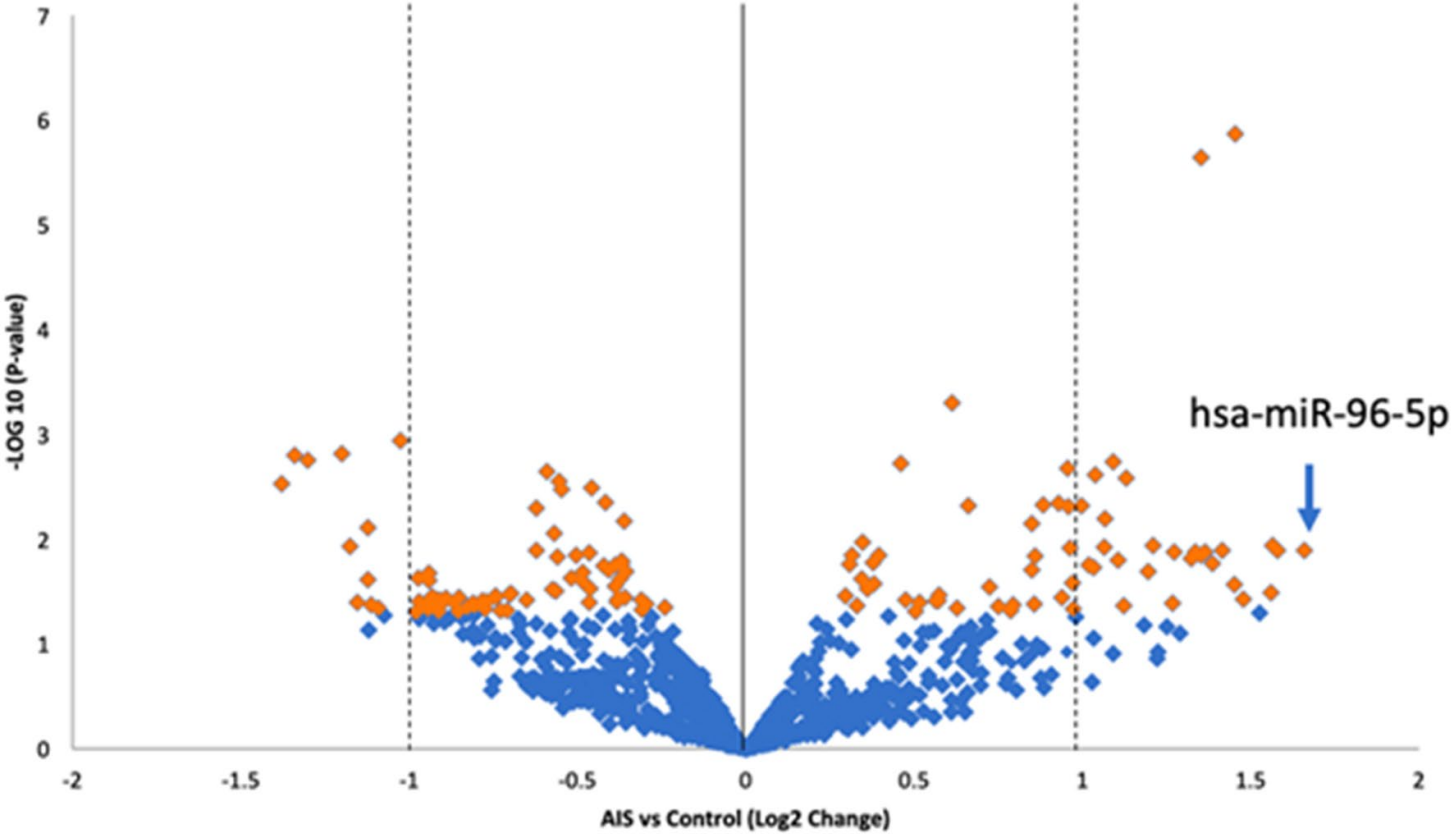
Differentially expressed miRNA candidates in iliac crest bone biopsies from AIS

**Figure 2:**
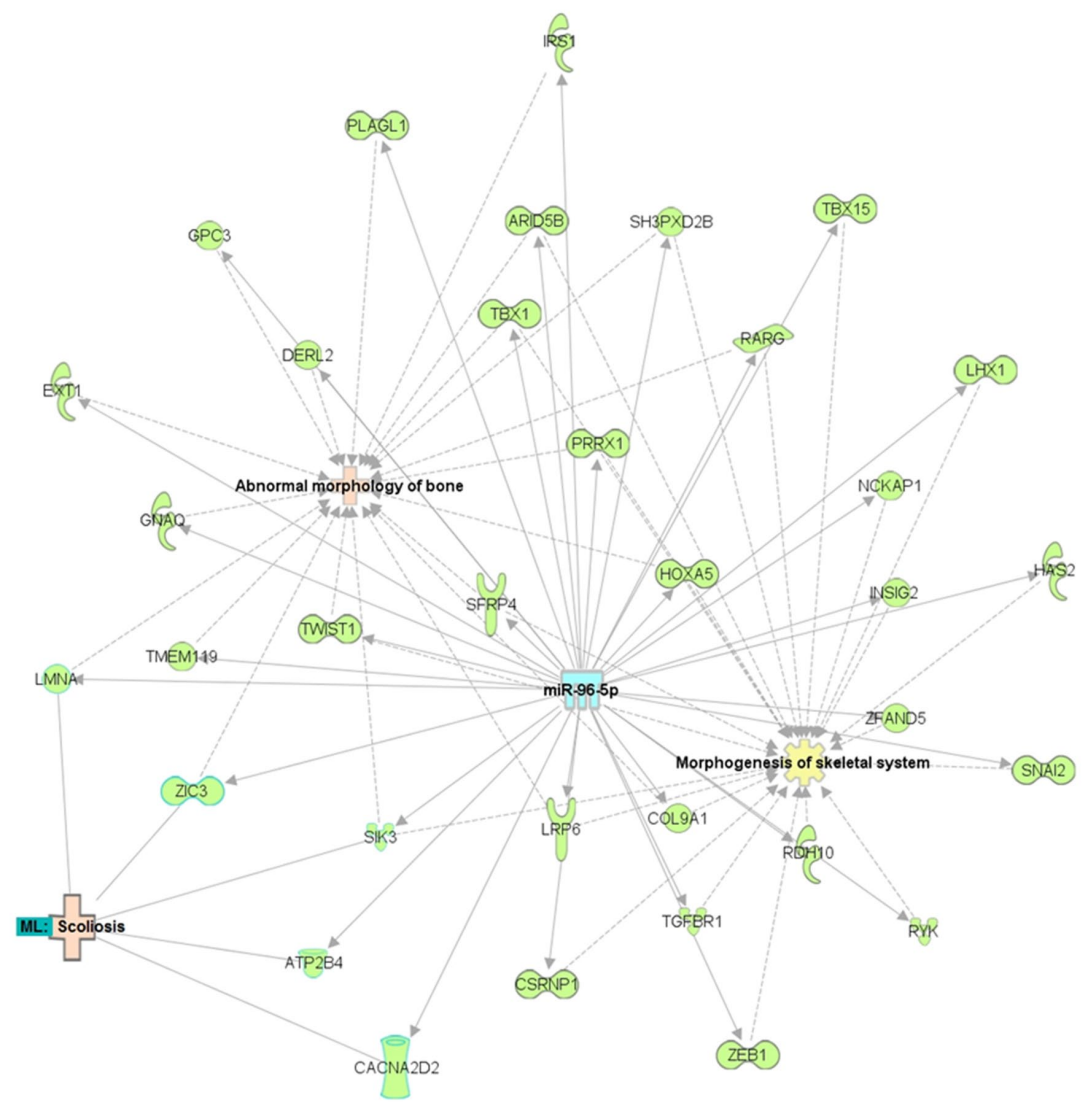
Predicted gene pathways and network of miR-96-5p. The miRNA is represented in blue; the genes that were predicted to interact with each other are in green; the diseases that were predicted to be associated with the miRNA or genes are in light pink; and the molecular and physiological functions are in yellow. The hybrid approach of IPA and manual curations was applied to construct the network of miR-96-5p.

A volcano plot (Fig. 1) was used to identify differentially expressed miRNAs in AIS bone. Thirty most differentially up- or down-regulated miRNAs in AIS bone tissues were summarized in Supplementary Table 2. Taken into consideration the fold change and statistical significance, miR-96-5p was selected due to its highest expression level among candidates with a 45.5-fold up-regulation when compared to non-scoliotic subjects. To get more insights into the mechanistic roles of our identified miRNAs in the molecular functions and their possible physiological roles in AIS pathogenesis, systematic gene pathway and network analyses were performed. Our analyses using the Ingenuity Pathway Analysis (IPA) software suggested that miR-96-5p targeting genes were playing more roles in abnormal morphology of bone and morphogenesis of skeletal system, and they were speculated to be associated with etiopathogenesis of scoliosis (Fig. 2).

### Higher plasma level of miR-96-5p in AIS

**Figure 3:**
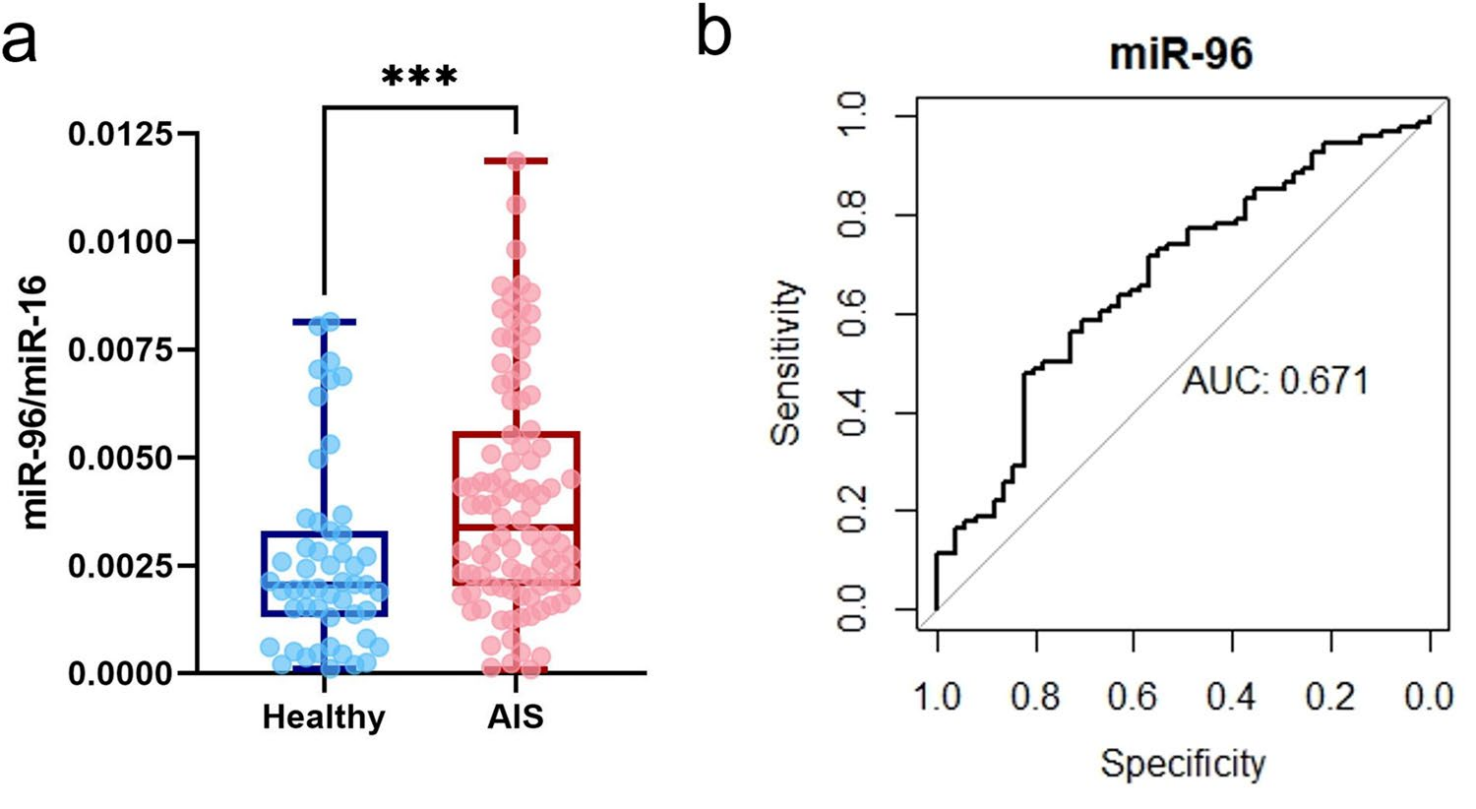
Plasma miR-96-5p levels in AIS and healthy girls. (**a**) Difference of plasma levels of miR-96-5p between AIS and healthy girls. Expression levels of miR-96-5p was normalized by plasma miR-16 levels. ***p  ≤  0.001. (**b**) Receiver operating characteristic (ROC) curve and AUC of plasma miR-96-5p level.

The expressions of miR-96-5p in plasma of 100 patients with AIS and 52 healthy girls were compared. Plasma levels of miR-96-5p were found to be significantly higher in the AIS group (p = 0.001) (Fig. 3a). The power of Mann–Whitney U test in detecting the difference between AIS and healthy girls was estimated to be 0.85. Area under the curve (AUC) analysis demonstrated discriminating potency of plasma miR-96-5p between AIS and healthy girls, suggesting reasonably good diagnostic potential of miR-96-5p with AUC of 0.671 (95% CI, 0.579–0.763) shown in Fig. 3b.

### Logistic regression models in distinguishing AIS patients from healthy controls

**Figure 4:**
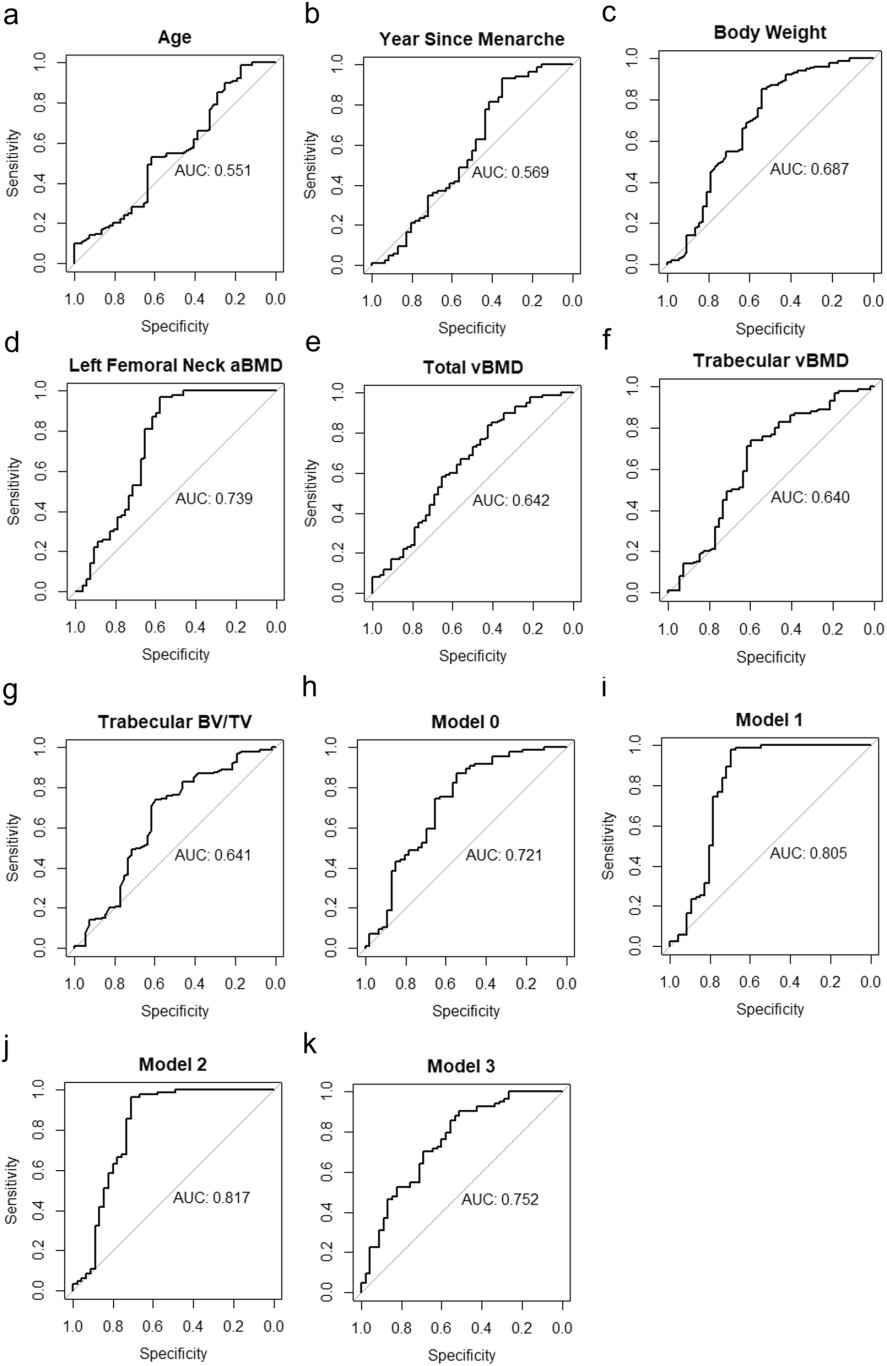
ROC curves and AUC of variables and logistic regression models.

**Figure 5:**
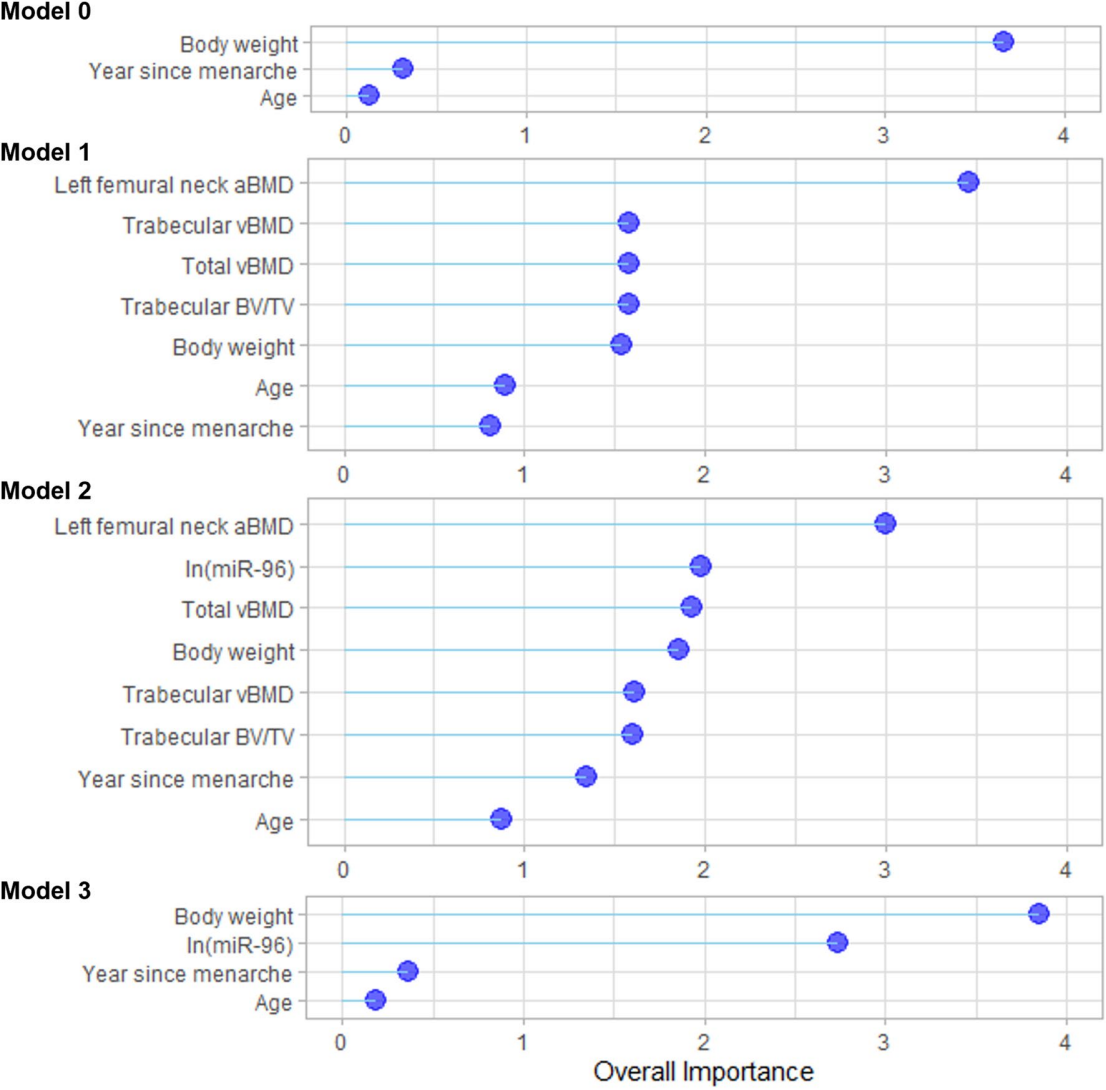
Variable importance in logistic regression models.

Logistic regression revealed that miR-96-5p along with body weight, femoral neck aBMD and total vBMD were significant predictors for the development of AIS (Table 3). The AUC for each variable was shown in Fig. 4. Given body weight, age and year since menarche are associated with curve progression and likely with onset of AIS^42–44^, these variables were included in model 0 as the default model for further comparisons (Model 0 in Table 3 and AUC in Fig. 4h). Model 1 was composed of bone quality parameters and classical risk factors (body weight, age and year since menarche) and explained 0.437 (Negelkerke R^2^) of the variances (Model 1 in Table 3 and AUC in Fig. 4i). Model 2 containing miR-96-5p and variables in Model 1 showed the highest Negelkerke R^2^ of 0.461 (Model 2 in Table 3) with AUC being 0.817 (95% CI 0.722–0.912, Fig. 4j). The addition of miR-96-5p increased the Negelkerke R^2^ of Model 0 with classical risk factors from 20.9% to 27.5% in Model 3 (Table 3). Meanwhile, the inclusion of miR-96-5p increased the AUC from 0.721 (95% CI 0.622–0.820) in Model 0 to 0.752 (95% CI 0.660–0.844) in Model 3 (Fig. 4k). The variable importance in each model was shown in Fig. 5.

**Table 3:** Logistic regression models to predict AIS.

	β	Standard error	Z value	P value	95% CI for β	
					Lower limit	Upper limit
**Model 0**						
Age	− 0.028	0.226	− 0.126	0.899	− 0.478	0.415
Year since menarche	− 0.059	0.187	− 0.316	0.751	− 0.430	0.310
Body weight	− 0.120	0.032	− 3.657	** < 0.001**	− 0.190	− 0.060
Constant	6.910	3.50	1.969	0.048		
Nagelkerke R-squared	0.209					
**Model 1**						
Age	0.234	0.262	0.890	0.371	− 0.276	0.762
Year since menarche	0.195	0.239	0.820	0.415	− 0.266	0.682
Body weight	− 0.064	0.042	− 1.540	0.124	− 0.150	0.013
Left femoral neck aBMD	− 10.500	3.040	− 3.460	** < 0.001**	− 16.934	− 4.944
Total vBMD	− 0.007	0.004	− 1.580	0.114	− 0.015	0.001
Trabecular vBMD	1.160	0.729	1.580	0.113	− 0.252	0.262
Trabecular BV/TV	− 1380.000	877.000	− 1.580	0.114	− 3155.600	308.790
Constant	96.10	4.100	2.350	**0.019**		
Nagelkerke R-squared	0.437					
**Model 2**						
ln (miR-96)	0.504	0.255	1.975	**0.048**	0.013	1.028
Age	0.234	0.269	0.871	0.383	− 0.287	0.776
Year since menarche	0.337	0.250	1.345	0.178	− 0.142	0.852
Body weight	− 0.078	0.042	− 1.854	0.063	− 0.166	0.0001
Left femoral neck aBMD	− 9.343	3.108	− 3.006	**0.0026**	− 15.907	− 3.652
Total vBMD	− 0.009	0.005	− 1.925	0.054	− 1.852	6.134
Trabecular vBMD	1.200	0.744	1.613	0.106	− 0.235	2.704
Trabecular BV/TV	− 1431.000	894.800	− 1.599	0.109	− 3238.296	296.308
Constant	11.900	4.341	2.740	**0.006**		
Nagelkerke R-squared	0.461					
**Model 3**						
ln (miR-96)	0.607	0.221	2.737	**0.006**	0.190	1.07
Age	− 0.044	0.237	− 0.189	0.850	− 0.516	0.421
Year since menarche	0.071	0.193	0.367	0.713	− 0.310	0.456
Body weight	− 0.127	0.033	− 3.853	** < 0.001**	− 0.197	− 0.067
Constant	10.850	3.988	2.721	**0.006**		
Nagelkerke R-squared	0.275					

Plasma level of miR-96-5p with log transformation, maximum Cobb angle at first visit, maturity (age, years since menarche), anthropometry (body weight, body height), left femoral neck aBMD, and bone qualities at distal radius (total area, cortical area, trabecular area, total vBMD, cortical vBMD, cortical thickness, cortical perimeter, trabecular vBMD, BV/TV, trabecular number, trabecular thickness, trabecular separation) were put into backward stepwise regression to select the important parameters for model 2. Bold text indicated p value < 0.05.

## Discussion

Significantly, higher expression levels of the miR-96-5p in AIS were shown in plasma samples from a retrospective case–control cohort. After excluding irrelevant or redundancy information that was leading to overfitting, a composite model composed of plasma miR-96-5p level, age, years since menarche and body weight was established and showed to have an AUC of 0.752 (95% CI 0.660–0.844) without bone qualities scan by DXA and HR-pQCT, indicating its potential to serve as clinically useful minimal invasive diagnostic biomarker for AIS.

The pathomechanism underlying the abnormal bone qualities has been one of the major research areas to understand the pathogenesis of AIS^1^. The findings of current genetic studies are far from reaching a consensus on biological contribution of individual single nucleotide polymorphisms (SNPs) to AIS pathogenesis. It appears that either single or multiple SNPs can only explain onset of AIS in small proportion of patients^45,46^. In view that AIS is a complex disease, the potential of epigenetics to explain its complexity and clinical heterogeneity represents a largely unexplored research field. Previous studies which relied on whole blood cells for the identification of prognostic biomarker candidates for AIS showed limited success^28,47^. Furthermore, given that DNA methylation patterns are often tissue specific, investigations focusing on DNA methylation alterations in whole blood cell of AIS patients are unlikely to be successful to decipher AIS pathogenesis^48–50^. Indeed, a large cohort study on 5515 individuals indicated a lack of association between epigenetic changes in whole blood and bone mineral density^51^. Therefore, we developed a more direct approach by investigating bone tissues in our discovery phase to identify miRNA candidates, which were likely to be more biologically relevant to the abnormal bone qualities in AIS.

Emerging evidence suggested that aberrant expression of several non-coding RNAs in AIS impaired cellular activities of mesenchymal stromal cells and osteoblasts/osteocytes^41,52,53^, which may account for the observed phenotypic changes affecting bone metabolism and bone qualities in AIS. In contrast to lncRNA, the stability of miRNA in biofluids renders its superiority for being biomarkers to reflect molecular alterations in complex diseases such as AIS^54,55^. The miR-96-5p identified in the present study had controversial effects on osteogenic activity. Yang et al. reported that miR-96 promoted the osteogenic ability of murine osteoblasts and bone marrow-derived mesenchymal stem cells^56^, and inhibits the proliferation of vascular smooth muscle cells^57^. Another publication showed that higher levels of miR-96 were associated with age-related bone loss^58^. Intravenous injection of agomiR-96 to 3-month-old mice induced defective osteogenesis by targeting osterix gene and the mice manifested low bone mass^58^. This in vivo evidence was supportive of observation in our current study that overexpression of miR-96-5p was associated with low bone mass and AIS in young Chinese adolescents. Our group previously reported that miR-145-5p impaired osteocytes activities and differentiation in AIS^41^ and it was a significant factor in a predictive model to predict whether the curve progressed to a Cobb angle of 40°^59^. Although the miRNA candidates were identified in bone tissues, it was of interest to provide functional evidence about their known and predicted downstream targets as well as their putative role in bone homeostasis.

There are several limitations of our current study. First, our validation cohort was cross-sectional by design. Our participants were skeletally immature, and we did not follow them until their skeletal maturity to determine the predictive potential of our miRNAs and models in prediction of the risk of spinal disease progression among a symptomatic population at an early stage. Therefore, a new prospective study with long-term follow-up is warranted. Secondly, expression of miR-96-5p was reported to be regulated by oestrogens (E2), implying the likelihood of a sexual dimorphism on this regard^60^. Indeed, one could rea sonably spe culate that the elevation of miR-96-5p was influenced by the hormonal rise, which could explain why girls are more predisposed to curve progression around puberty than boys. The present study only validated the abnormal expression of miR- 9 6-5p in girls and future validations in both females and males are required to clarify whether expression of miR-96-5p is abnormal in male AIS patients and whether it is differe nt between genders. Third, although abnormal miRNAs that were identified in bone tissue in AIS have been validated in our previous work (miR-145-5p) and the present study (miR-96-5p), a c ombination of multiple biomarkers from different omics layers is warranted in future work to achieve an optimal effectiveness in early detection or prognostication for AIS.

In summary, this study proposed a predictive model for AIS using pathology-associated clinical features such as low bone mass and aetiology-associated circulating miR-96-5p levels, which possessed the ability to distinguish AIS patients from healthy controls. This study addressed the outstanding clinical question for a reliable diagnosis system of AIS at the early presentation of AIS, which has important clinical implications for clinical decisions to prevent onset and to avoid over-treatment and frequent radio-exposure.

## Methodology

### Study populations

Patients undergoing surgery were recruited for microarray assay. Trabecular bone tissues were intraoperatively collected from iliac crests of AIS patients who underwent spinal fusion surgery as a part of taking autograft to enhance bony fusion. Trabecular bone tissues from non-scoliotic controls were collected from trauma cases who needed iliac crest bone tissues during orthopedics surgery. To investigate the clinical implication of circulating miRNAs, patients were recruited in a case–control cohort. Anthropometrical parameters including standing height, sitting height, body weight and arm span were measured with standard protocols. AIS was diagnosed clinically by at least two senior orthopedic surgeons and radiologically with standing full-spine posteroanterior  (PA) X-ray. HR-pQCT measurement, DXA measurement and full-spine PA X-ray for Cobb angle of the major curve were performed within a month before or after the date when blood samples were taken. In the case–control cohort, one hundred AIS girls were recruited from the scoliosis clinic in Prince of Wales Hospital, Hong Kong. Fifty-two aged matched heathy girls were recruited randomly from local secondary school as control. Exclusion criteria included subjects with congenital deformities, neuromuscular diseases, autoimmune disorders, endocrine disturbances or other medical conditions that affecting bone metabolism. E thical approval in accordance with Declaration of Helsinki was obtained from Clinical Research Ethics Committee of the University and Hospital (CREC No. 2016.567). Written informed consent was ob tained from all the subjects an d their legal guardians before the examinations and measurements.

### BMD and bone qualities assessment

aBMD of bilateral femoral necks was measured by DXA (XR-46; Norland Medical Systems, Wisconsin, USA). Due to the bias caused by axial vertebral rotation in AIS, aBMD of the spine measured by DXA should not be used in AIS^61^. The detail of DXA measurement was presented in our previous study^32^. The 3-dimentional assessment of bone qualities in the non-dominant d istal radius was measured with HR-p QCT (XtremeCT I, Scanco Medical, Brüttisellen, Switzerland) according to the standard protocols^62^. Reference line was marked at the most proximal point of the inner aspect of the growth plate. One-hundred-ten computed tomography slices with a resolution of 82 μm were obtained from a segment spanning 9.02 mm starting from 5 mm proximal to the reference line.

### miRNA microarray on bone biopsies

Total RNA was extracted from iliac crest bone biopsies from 4 AIS and 4 non-scoliotic subjects with TRIZOL (Life technologies, California, USA) based on manufacturer’s instruction. RNA integrity was tested with Agilent Bioanalyzer 2100 (Ag ilent Technologies, Palo Alto, California, USA) to ens ure satisfactory RNA integ rity and quali ty before microarray a ssay. Differentially expressed miRNAs were identified by Agilent expression array-Human miRNA 8 × 60k (Agilent Technologies, Palo Alto, CA, USA) with 100 ng of total RNA. Agilent m icroarray could ident ify all matu re miRNA sequences i n the latest miRbas e (version 21.0) w ith hi gh sensiti vity and wide coverag e. Data processing was conducted with GeneSpring station (version 12.6).

### Construction of gene pathways and networks targeted by dysregulated miRNAs in AIS

The potential targets of miRNAs of interest, includ ing genes, molecular and physiological functions in AIS pathogenesis were primarily identified through a comprehensive literature review and manual curations. The connections (interactions) of the miRNAs and their targets were constructed based on the Ingenuity Knowledge Base using the Ingenuity Pathway Analysis (IPA) software (QIAGEN Inc. software version 51,963,813).

### Plasma miRNA level assay

Peripheral venous blood samples from subjects were collected by phlebotomists with standard technique. Blood was centrifuged at 4 °C and 1500×*g* for 10 min. Plasma was aliquoted and stored at − 80 °C f or further analysis. Thaw-freeze cycle was avoided in case of degradation. Isolation of circulating miRNAs from plasma was performed with miRNeasy plasma advanced k it (217204, Qiagen , Venlo, The Netherlands) according to manufacturer’s protocol. cDNA library was constructed, and target miRNA expression levels were measured with TaqMan^@^ advanced miRNA assay (Life Technologies, California, USA). miR-16 was chosen as an internal reference after evaluating its stability in BestKeeper (http://www.gene-quantification.de/bestkeeper.html/).

### Statistical analysis

The normality of data was tested by Shapiro–Wilk test. Normal data was presented as mean ± SD and the other was presented as median (lower quartile, upper quartile). To compare the parameters between AIS and non-scoliotic controls, independent sample t test was used for the normal data, and the Mann–Whitney U test was us ed for the skewed data. Analysis of covariance (ANCOVA) analysis was used to compare miRNA levels between AIS and healthy girls with adjustment to age. Backward stepwise regression was used to determine whether the plasma miRNA is a important predictor in classifying AIS from non-scoliotic subjects with adjustment for bone maturity, body size, bone qualities by HR-pQCT and aBMD by DXA. In the logistic regression, plasma miRNA levels were transformed with natural log transformation. The factor was dichotomised with criteria shown as follows: 1 as logistic score ≥   0.5 and 0 as logistic score < 0.5. ROC curves and the binary classification tables were used to study sensitivity and specificity. Variable importance was estimated using a method described by Gevery et al.^63^ P-values < 0.05 (two-tailed) indicated statistical significance. Data collection and analyses were performed by two blinded investigators independently. Analyses were per formed using R (version 4.0.0, The R Foundation, Vienna, Austria). Effect size of AIS status on expression of miR-96-5p was calculated by dividing the difference between means pertaining to AIS and healthy girls by SD of the whole validation cohort. Power of statistical testing were estimated by G*Power (ve rsion 3.1.9.6, Kiel University, Germany) with α being 0.05.

### Ethics declarations

Ethical approval in accordance with Declaration of Helsinki was obtai ned from Clinical Research Ethics Committee of the University and Hospital (CREC No. 2016.567). Written informed consent was obtained from all the subjects and their legal guardians before the examinations and measurements.

## Supplementary Information

Supplementary Information.

## Data Availability

The data that support the findings of this study are available from the corresponding author upon reasonable request.
