# Supplementary material for: Upregulation of microRNA-96-5p is associated with adolescent idiopathic scoliosis and low bone mass phenotype
Source: Sci Rep. 2022 Jun 11;12:9705. doi: 10.1038/s41598-022-12938-3 (PMC9188568; doi:10.1038/s41598-022-12938-3)
Supplement: Supplementary file 1 — Supplementary Information. [file 41598_2022_12938_MOESM1_ESM.pdf]

Supplementary Table 1 Diagnosis of non-scoliotic controls

Case No.	Diagnosis	Sex
Control 1	Lumbar vertebral fracture	Male
Control 2	Lumbar spondylolisthesis	Male
Control 3	Lumbar spondylolisthesis	Female
Control 4	Left scapula osteomyelitis	Male

Supplementary Table 2 List of microRNA candidates with highest fold change in microarray analysis: Up indicates higher level in AIS

miRNA candidate	P value	Fold change	Expression	mirbase accession No
hsa-miR-96-5p	0.012711299	45.50291	up	MIMAT0000095
hsa-miR-374a-5p	0.012799246	38.01273	up	MIMAT0000727
hsa-miR-582-5p	0.011440388	36.68986	up	MIMAT0003247
hsa-miR-144-5p	0.032286912	36.230274	up	MIMAT0004600
hsa-miR-20a-5p	0.050941724	33.674187	up	MIMAT0000075
hsa-miR-18a-5p	0.03714784	30.129683	up	MIMAT0000072
hsa-miR-4449	1.34E-06	28.421408	up	MIMAT0018968
hsa-miR-20b-5p	0.027044091	28.237808	up	MIMAT0001413
hsa-miR-454-3p	0.012692338	25.976322	up	MIMAT0003885
hsa-miR-363-3p	0.016952466	24.380987	up	MIMAT0000707
hsa-miR-4659a-3p	0.00293014	23.866352	down	MIMAT0019727
hsa-miR-3663-5p	2.29E-06	22.5057	up	MIMAT0018084
hsa-miR-7-5p	0.014309563	22.446573	up	MIMAT0000252
hsa-miR-6865-3p	0.001584507	21.8517	down	MIMAT0027631
hsa-miR-215-5p	0.013323674	21.688925	up	MIMAT0000272
hsa-miR-194-5p	0.015177659	21.091337	up	MIMAT0000460
hsa-miR-6851-3p	0.001749121	20.048632	down	MIMAT0027603
hsa-miR-26b-5p	0.07954026	19.460506	up	MIMAT0000083
hsa-miR-335-5p	0.013214523	18.672852	up	MIMAT0000765
hsa-miR-18b-5p	0.041104604	18.500933	up	MIMAT0001412
hsa-miR-19a-3p	0.06827171	17.815714	up	MIMAT0000073
hsa-let-7g-5p	0.120146334	16.775314	up	MIMAT0000414
hsa-let-7f-5p	0.13683109	16.696346	up	MIMAT0000067
hsa-miR-223-3p	0.011423348	16.236929	up	MIMAT0000280
hsa-miR-4715-5p	0.001508573	15.8491955	down	MIMAT0019824
hsa-miR-183-5p	0.020198133	15.639239	up	MIMAT0000261
hsa-miR-98-5p	0.06598188	15.226245	up	MIMAT0000096
hsa-miR-6856-5p	0.011706335	15.014675	down	MIMAT0027612
hsa-miR-3190-3p	0.039877497	14.246491	down	MIMAT0022839
hsa-miR-451a	0.002605507	13.467289	up	MIMAT0001631
